# Seeking Amyloidosis Very Early: Free light Chain Differentials and IGLV Gene Use as Screening Variables for Light-chain Amyloidosis in λ Monoclonal Gammopathies

**DOI:** 10.31488/bjcr.193

**Published:** 2024-05-23

**Authors:** Ping Zhou, Mahesh M Mansukhani, Raymond Yeh, Jiesheng Lu, Hongai Xia, Lahari Koganti, Jiuhong Pang, Denis Toskic, Stephanie Scalia, Xun Ma, Nancy Coady Lyons, Teresa Fogaren, Cindy Varga, Raymond L Comenzo

**Affiliations:** 1. The John Conant Davis Myeloma and Amyloid Program, Tufts Medical Center, Boston, MA, USA; 2. Columbia University Laboratory of Personalized Genomic Medicine, Department of Pathology & Cell Biology, Columbia University Medical Center, New York, NY, USA; 3. Division of Hematology-Oncology, Department of Medicine, Tufts Medical Center, Boston, MA, USA; 4. Atrium Health Levine Cancer Institute, Charlotte, NC, USA

**Keywords:** AL amyloidosis, IGLV genes, next generation sequencing, monoclonal gammopathies

## Abstract

**Background:**

Early diagnosis of systemic light-chain amyloidosis (AL) is needed because 25% of patients die within months of diagnosis. In patients with monoclonal gammopathy of undetermined significance (MGUS) or smoldering multiple myeloma (SMM) of the λ isotype, we explored the use of 2 screening variables: a free light chain difference of 23mg/L between λ and k and presence of IGLV genes that occur more frequently in AL.

**Methods:**

Patients contacted us and we sent HIPAA release and consent forms for discussion by phone. Their physicians were not involved. We enrolled patients with λ MGUS or SMM who met the FLC criteria with no prior biopsies showing amyloid. They sent us blood or marrow specimens for IGLV gene amplification by RT-PCR; we also assessed the feasibility of next generation sequencing (NGS) for IGLV genes. We informed patients and their physicians of results suggesting further evaluation for AL.

**Results:**

We enrolled 21 patients, 19 SMM and 2 MGUS, receiving blood (n=21) or marrow (n=5) specimens. We identified IGLV genes in 86% (18/21) of cases. Four of the 18 IGLV genes were not AL-related and 3 of these 4 progressed to myeloma requiring therapy; the 4th was screened for amyloid and was negative. Fourteen patients with AL-related genes had comprehensive evaluations and two with SMM had AL. RT-PCR and NGS identified the AL-related LV2–14 in those two and also the monoclonal IGLV genes from all of the marrow but not the peripheral blood samples.

**Conclusion:**

We concluded that these variables may be useful in screening for AL in λ MGUS and SMM patients and acquired support for a small multi-center study employing marrow samples only.

## Background

In a retrospective study employing pre-diagnostic specimens from members of the US Armed forces subsequently diagnosed with AL, investigators at Walter Reed showed that for a decade prior to diagnosis 80% had monoclonal serum free light chain (FLC) abnormalities and that, when diagnosed with AL, 55% had cardiac involvement [1]. Delays in diagnosis of AL occur even in patients with MGUS or SMM, known risk factors for the disease, in the care of hematologists [2,3]. Early diagnosis is critical given the frequency of cardiac involvement and current availability of effective therapies [4–6].

Numerous monoclonal disorders display biased repertoires of immunoglobulin variable region gene use [7–10]. In AL there is λ isotype predominance with a κ-to-λ case ratio of 1-to-3 and biased λ IGLV gene use [11,12]. Given this frequency bias and the results of the retrospective study in US service members, we hypothesized that clonal expression of one of the λ genes frequently found in AL and a difference between involved and uninvolved FLC > 23mg/L, as in 85% of AL cases in US service members prior to diagnosis, could presage risk of AL. We conducted a single-center internet-based and patient-driven IRB-approved pilot study to test the feasibility of using these 2 variables to screen for AL in patients with λ MGUS or SMM. Specimens were sent to us by eligible patients for IGLV gene identification. Upon gene identification we notified patients and their physicians of our findings, suggesting a further evaluation for AL especially in those with an AL-related IGLV gene. We subsequently sent de-identified specimens to a clinical precision medicine laboratory for next generation sequencing (NGS) identification of IGLV genes to test the feasibility of creating a laboratory developed NGS test in λ monoclonal gammopathies. We now report the results of this preliminary internet-based pilot study (NCT02741999).

## Patients and Methods

### Patients

The Health Sciences Institutional Review Board (HS IRB) at Tufts Medical Center approved our single-center study titled “A Diagnostic Screening Trial Seeking AL Amyloidosis Very Early” (Approval number: 12016). Recruitment started on April 4, 2017 and ended on April 4, 2020. Participants were required to provide written informed consent in order to participate. This study was minimally supported with philanthropy. Only specimens drawn at times of clinically indicated testing were permitted by our IRB to be sent to us; physician collaborators for specimen procurement at outside institutions were not permitted. Patients were responsible for having samples obtained at times of clinical evaluation and for sending them to us overnight in kits we provided. We initially projected that we might enroll up to 200 patients before seeing a signal; that was not the case as we show.

Based on internet advertising of the study, patients contacted us by e-mail and telephone. We then sent HIPAA release and protocol consent forms directly to them that they discussed with us by phone, completed and returned. To be eligible, patients had to have λ isotype MGUS or SMM with a dFLC > 23mg/L, a κ/λ ratio below normal and no prior biopsies showing amyloid. Patients were enrolled after review of their medical records. Their physicians were not involved in screening or obtaining specimens, except to provide phlebotomy and to give patients samples of marrow aspirates, when marrow studies were clinically indicated, to send to us.

Then, patients shipped specimens to us in biologic hazard kits by prepaid overnight delivery either blood (PB) or marrow (BM). No preliminary study-related testing was allowed by our IRB.

### IGLV Genes

In analyses of IGLV genes in AL and MM, the frequencies of specific IGLV genes have been identified [12,13]. The 9 IGLV genes considered AL-related were LV1–40, LV1–44, LV1–51, LV2–14, LV2–23, LV3–1, LV3–19, LV3–21, and LV6–57, exhibiting a significant frequency bias compared to myeloma cases with the same IGLV genes in AL-Base. Initially we identified 5 IGLV genes as relevant to AL but with additional information we expanded to the 9 of 33 IGLV germline genes on chr 22q11.2 as relevant; these 9 account for 95% (N=395) of the AL IGLV sequences in the Boston University database of IGLV sequences from 417 systemic AL λ-type patients and for 83% (N=248) from 340 myeloma λ cases (P<<0.01, two sided χ2; odds ratio 6.661 with 95% CI 4.074 to 10.89) [12,14]. We used these 9 genes to inform this preliminary pilot study, calling them relevant to AL λ-type. Obviously these donor genes are found in both AL and MM and therefore IGLV findings do not represent a single indicator of likelihood of AL.

As possible variables in a hypothetical likelihood algorithm comprised of multiple variables designed to estimate risk of AL in individuals with λ SMM or MGUS, however, the presence of an AL-related IGLV gene and of the FLC criteria may be useful.

### Specimen management

Specimens were sent to us by overnite delivery in green top tubes. Mononuclear cells from PB or BM were separated with Lymphoprep (StemCell Technologies; Vancouver, CA). CD138-microbead selection was then performed with the Mini-MACS (Miltenyi Biotec; Somerville, MA, USA) as per the manufacturer’s instructions. RNA extraction from CD138-selected cells was performed with the RNeasy Plus Mini-Kit (Qiagen; Hilden, Germany) and cDNA synthesized with the ThermoScript RTPCR System (Invitrogen; Carlsbad, CA, USA) for storage at −80° C [15,16].

### RT-PCR and NGS

PCR was conducted with Taq DNA Polymerase (Invitrogen) and the amplicons identified and prepared for core lab sequencing with Wizard SV Gel and PCR Clean-UP System (Promega; Madison, WI, USA). Primers used for PCR were as previously described for V1, V2, V3 and V6 λ families [17]. Each specimen was subject to multiple amplifications and bands were selected for sequencing from several separate PCR experiments to confirm the reproducibility of the amplified sequence. With each confirmed sequence we then identified the corresponding IGLV gene in the ImMunoGeneTics database (IMGT, www.imgt.org). Patients and their physicians were notified of the IGLV gene in each case and, if it was one of the 9 deemed relevant to AL λ-type, we strongly advised further evaluation for AL with fat pad aspirates stained with Congo red and standard urine and blood testing including cardiac biomarkers.

Subsequently, we sent de-identified specimens of cDNA from CD138-selected cells to Columbia University for NGS evaluation. Nine variable region and two constant region primers were used with the Q5 high-fidelity DNA polymerase (NEB, Ipswich MA) [18]. The resulting PCR products were sheared by ultrasonication and, following end-repair and adapter ligation, sequenced on a MISeq (Illumina, San Diego CA) using a 300-cycle paired-end flow cell. Approximately 500,000 reads were obtained per sample after demultiplexing.

Fastq files were converted to FASTA sequences and these were mapped for IGLV gene segments using IGMT/HIV-Quest to obtain clonal reads.

### Statistics

Statistical analyses were performed with Graphpad Prism (GraphPad, San Diego, CA).

## Results

### Twenty-one patients from 18 States enrolled

Despite IRB and funding limitations, requiring us to involve only patients not physicians and to employ specimens obtained solely at clinically indicated times and sent to us by the patients, 25 patients requested screening between 2016 and 2020 in response to internet advertising and postings on advocacy group websites; 4 patients (from Kentucky, Mississippi, New Hampshire and Massachusetts) were screened out because either or both dFLC or κ/λ ratio did not meet criteria. We enrolled 21, 19 with SMM and 2 with MGUS, from 18 states in the USA, whose baseline characteristics are summarized in Table 1. Complete patient data including Genbank accession numbers for all of the IGLV genes are provided in the Harvard Dataverse [19]. We stated at baseline that we could accrue up to 200 subjects but closed this pilot study to accrual in mid-2020 given a stronger signal than anticipated and also given approval of a funded multicenter study (NCT04615572; opened 08–2020); however, we followed by phone the 21 subjects on this pilot study for outcomes until 7/1/2022.

At screening, we talked with the patients, confirmed their lack of symptoms as consistent with medical records sent to us under HIPAA release including physician notes, laboratory studies, reports of radiographic and other studies, and pathology reports. We then sent to the enrolled patients premade kits containing green top tubes, packing material and prepaid overnite delivery forms with instructions. No patient we enrolled had been diagnosed with AL, had had prior biopsies that showed amyloid or was being treated for MM at the time of enrollment.

### Specimens and RT-PCR results

From these 21 patients we received PB (n=21, each about 15ml) or BM (n=5, each about 5ml) specimens. With hematocytometer trypan blue manual counts, PB specimens contained a median of 7.6×10^6^ MNC (1.3–24) and BM specimens contained a median of 9.7×10^6^ MNC (8.2–21). By RT-PCR with cDNA from CD138-selected cells we identified an IGLV gene in 86% (18/21) of cases, failing in 3 cases, 2 SMM and 1 MGUS. Four of the 18 IGLV genes (LV1–47, LV2–11, LV3–25, LV4–69) were not AL-related and 3 of these 4 patients progressed to MM requiring therapy without a finding of amyloid. Fourteen patients with AL-related genes had comprehensive local evaluations and two were found to have AL with LV2–14. Both were being followed for SMM and both had physicians aware of the IGLV findings; these patients had additional tests including GI biopsy in one case and heart biopsy in the other (based on MRI findings) that showed amyloid. In both cases, diagnosis was confirmed by mass spectroscopy [20]. Table 2 contains details on these 2 patients and excerpts from the biographical accounts they provided to the National Institute of Aging (R21-AG070502-01). Three others in the group of 14 progressed to MM requiring therapy.

### Patient voices

#### Patient One

I cannot thank you enough for the lifesaving outcome your SAVE trial had on my life. I contacted my doctor and he ordered some preliminary tests. Nothing was discovered, but because this disease is so serious we wanted to be safe not sorry so I had a fat pad aspirate and bone marrow biopsy in June 20XX. A mass spectrometry test revealed amyloid deposits in both samples. In September 20XX a biopsy of my stomach showed amyloid deposition. The transplant was performed on an outpatient basis. The ease of the transplant, chemo, and recovery were all due to early diagnosis through the SAVE trial.

#### Patient Two

On April XX, 20XX my results showed a germline gene donor IGLV2–14. I had additional blood work, a urine test and a fat pad biopsy done with negative results. On October XX, 20XX, I had a mild cardiac episode that landed me in the hospital for an echocardiogram and a nuclear stress test. Nothing alarming was found from the tests, but when I raised the issue of my gene identified by the SAVE Trial, the attending doctor said I should have a cardiac MRI. The cardiac MRI did show a small amount of deposits in my heart. Next the cardiologist ordered a heart biopsy that confirmed that the deposits were AL amyloidosis. I now can be counted among those patients who have participated in the SAVE Trial whose lives have actually been saved by this research.

### NGS Results

Subsequently, as part of this pilot study’s feasibility effort, we performed NGS for IGLV genes using 20μL of cDNA from CD138-selected cells from 20 of the cases in blinded fashion in a CLIA-certified precision medicine laboratory. NGS identified IGLV genes in 90% (18/20) of cases (BM=5, PB=13), failing in 2 PB cases. With cDNA from bone marrow CD138-selected cells, RT-PCR and NGS gave identical monoclonal results. There were no BM cases with multiple clones by NGS. As the following data indicate, bone marrow was the better source of cDNA for clonal genes than PB but whether BM MNC or CD138-selected cells are better for NGS with marrows containing less than 20% clonal plasma cells remained an open question [21].

For the 18 cases, median clonal reads were 58,825 (IQR 41,692–86,946). There was no difference in clonal reads between PB and BM samples. With cDNA from PB CD138-selected cells, 7 of the 13 cases (SMM=6, MGUS=1) demonstrated polyclonal findings with 2 (n=4), 4 (n=2) and 10 (n=1) different clones respectively. The number of reads varied; in 1 case with 2 clones (LV3–1, LV3–21) the ratio of reads was 2:1 with RT-PCR identifying a different gene, LV3–19. All 3 of these IGLV genes are AL-related and this patient was screened for AL and was negative. In the other 3 cases, RT-PCR failed to identify a clone in 2 and in the third identified a different clone than those identified by NGS. In one case with 4 clones, RT-PCR identified the AL-related LV2–14, the second place NGS clone; the rest of the clones were also AL-related (LV2–23, LV3–1, LV1–51). The patient was screened for AL and was negative. In the other case with 4 clones, RT-PCR identified LV3–19 while NGS identified clones with reads as follows: LV1–44 60,698, LV1–51 19,729, LV3–19 6,398, and LV3–21 5,739. Again all 4 were AL-related and the patient was screened for AL and was negative. RT-PCR gave no results in the case that was a polyclonal MGUS with 10 clones. With cDNA from PB CD138-selected cells in the 6 other PB cases, RT-PCR and NGS gave identical results in 5 and different results in 1. Six of the 18 IGLV genes identified by NGS were not AL-related (LV1–47 (n=2), LV2–11, LV3–25, LV7–46, LV10–54) and 2 failed RT-PCR. The other 10 patients had AL-related genes including the 2 with LV2–14 found to have AL and the remaining 8 who were screened for AL but were negative. Clearly bone marrow and not PB was the better source of cDNA for the identification of clonal genes but whether BM MNC or BM CD138-selected cells are better for NGS using marrows with fewer than 20% clonal plasma cells remained an open question [21].

### Follow-up

By 7/1/2022, 7 patients with SMM had gone on to receive therapy, 6 for MM and 1 for a brain tumor. These patients were a median of 20 months (12–60) from the diagnosis of SMM when they enrolled on this study; 3 had AL-related IGLV genes (LV2–14 (n=2), LV3–19). Overall by then, 9 of 21 (MM=6, AL=2, brain tumor=1) or 43% progressed to therapy after enrolling on this pilot study.

## Discussion

This study was not a standard diagnostic study but rather a preliminary unfunded pilot study. Our IRB did not allow PB draws or BM procedures to be performed to provide specimens for this study. All specimens had to be obtained at times of clinical procedures, either PB draws or BM studies. The goals of the study were to determine the feasibility of employing an internet-based patient-driven model to identify whether FLC criteria and clonal IGLV genes in patients with λ MGUS or SMM could be useful as screening variables for risk and presence of AL and to assess the feasibility of performing NGS with patient cells. If feasible, NGS could provide a basis for the creation of a laboratory developed test that could be useful for screening; RT-PCR was not being considered for progression to a laboratory developed test.

Our results demonstrate the feasibility of some features of the study: the workability of the patient-driven model, the reliability of overnite shipping of blood and marrow cells, the successful identification of IGLV genes from limited samples of BM, and the effective conduct of NGS for IGLV gene specification with BM specimens. Not surprisingly BM was a much more reliable source of clonal cells than PB; with the BM samples results with RT-PCR and NGS were 100% concordant and monoclonal. We attribute the discordant results with PB to the potential spectrum of CD138+ cells in PB, including differentiating B-cells and plasmablasts, particularly in patients with polyclonal gammopathy, as well as clonotypic cells [22–24].

We obtained a strong signal from this pilot study because we identified 2 undiagnosed cases of AL associated with the LV2–14 gene, representing 11% of those being followed for SMM (2/19). These two patients achieved complete hematologic responses with therapy, one with stem cell transplant and the other on the Andromeda trial (NCT03201965). Based on the results of this pilot study we were able to fund a multi-center study employing research bone marrow specimens; therefore, we closed this study at 21 subjects having shown that undiagnosed cases of AL could be identified in patients with λ monoclonal gammopathies.

It was of interest that 14 of 21 patients, 13 with SMM and 1 with MGUS, had genes identified by RT-PCR that were AL-related. There are several implications of this finding. One is that the FLC criteria we employed may have had a selective effect. The second is that clonal IGLV germline gene utilization in AL is less restricted than we assume, and the third is that the possible risk of AL in patients with SMM may be more salient than we conventionally think. One in seven patients diagnosed with MM is asymptomatic with SMM. Over years, based on Olmstead County data, 2% of patients with SMM progress to AL and 57% to MM, and of those who progress to MM 10% subsequently develop AL [25]. It is worth keeping in mind that the current practice pattern in SMM pays limited attention to risk of AL despite the fact that perhaps 12% of SMM patients may eventually get AL.

In addition to the possible utility of both dFLC > 23mg/L and the presence of an AL-related IGLV gene, an additional variable involving the cytogenetic findings in AL plasma cell clones may be useful in the construction of a likelihood algorithm. In SMM 23% of patients are cyclin D1-positive with t(11;14), 30% have gain 1q and 12% are hyperdiploid [26–29]. In a series of 133 AL patients whose marrow plasma cells were evaluated for clonal cytogenetic abnormalities, 83 (62%) had t(11;14), 35 of 130 (27%) had gain 1q and 24 of 125 (19%) were hyperdiploid [31]. In another series of 140 AL patients, 59% had t(11;14), 20% had gain 1q and 13% were hyperdiploid. In a series of 401 AL patients whose clonal plasma cells were evaluated at Mayo Clinic, 81% had abnormal fluorescent-in-situ findings, including 43% with t(11;14) and 12% with hyperdiploidy; gain 1q was not assayed [31]. In a series of 113 AL patients, the majority of whom had > 10% clonal plasma cells (that is, SMM), 39% had t(11;14), 22% gain 1q and 16% were hyperdiploid.(72) On average, then, 51% of AL cases are t(11;14) and at least 23% are gain 1q. The t(11;14) translocation, deleting the heavy chain locus, likely accounts for the high frequency of FLC-producing clones in AL.(20) It is possible then that FLC criteria, IGLV gene identification and the ascertainment of t(11;14) and gain 1q in clonal plasma cells may be useful variables in an algorithm for screening SMM patients for risk of AL.

## Conclusion

In conclusion, we show that in patients with λ MGUS or SMM the use of two variables, a dFLC > 23mg/L and the presence of an AL-related IGLV gene, may enable early diagnosis of AL. We also show that NGS works effectively with bone marrow as opposed to peripheral blood CD138-selected cell cDNA. The development of an approach to identifying AL and risk of AL that enables earlier diagnosis prior to the stage of significant organ damage is a critical unmet need. Combining data from testing of monoclonal gammopathies into a quantitative algorithm will require a collaborative effort to procure a large sample of cases and to monitor outcomes prospectively.

Additional efforts currently underway to enable early diagnosis include novel radiographic and protein-based techniques that have promise but in contrast may require significant commercial support and regulatory evaluation [32,33]. Construction of a likelihood algorithm employing data from standard-of-care and NGS evaluations is compatible with those approaches should they come to fruition.

## Figures and Tables

**Table 1.** Baseline Characteristics

Characteristic	Patients (n = 21)
Age, years	
Median (range)	57.5 (39–75)
Male, n (%)	5 (23)
Months from diagnosis of λ SMM (n=20)	15.5 (1–99)
Months from diagnosis of λ MGUS (n=2)	34, 58
λ FLC (mg/L) (5.7–26.3)	99.2 (26.3–1420)
κ/λ Ratio (0.26–1.65)	0.09 (0.01–0.73)
M-protein (g/dL)	1.18 (0–3.7)
Immunoglobin heavy chain isotype, n (%)	
IgG	15 (71)
IgA	6 (29)
Creatinine (0.6–1.2 mg/dL)	0.8 (0.64–1.3)
Albumin (3.5–5.5 g/dL)	4.2 (3.6–4.6)
Alkaline Phosphatase (20–140 IU/L)	72 (34–111)

**Table 2.** Two Patients Diagnosed with AL with LV2–14

Characteristic	Patient One	Patient Two
Age/Gender	56/F	58/F
Months with SMM	9	99
λ FLC (mg/L) (5.7–26.3)	133	124.4
κ**/**λ Ratio (0.26–1.65)	0.09	0.05
M-protein (g/dL)	IgGλ 1.7	IgAλ 0.48
NT-proBNP (pg/mL)	221	412
Troponin I (ng/mL)	0.01	0.03
Creatinine (0.6–1.2 mg/dL)	0.9	0.69
Albumin (3.5–5.5 g/dL)	3.8	3.8
Alkaline Phosphatase (20–140 IU/L)	43	64
Biopsy Sites Positive	Fat, marrow, GI	Heart

## Data Availability

Complete patient data including Genbank accession numbers for all of the IGLV genes are provided in the Harvard Dataverse. (19) Patient materials are no longer available.

## Abbreviations

AL: Systemic light-chain amyloidosis
BM: bone marrow
dFLC: difference between involved and uninvolved free light chains
FLC: free light chains
IGLV: immunoglobulin lambda variable
MGUS: monoclonal gammopathy of undetermined significance
MM: multiple myeloma
MNC: mononuclear cells
NGS: next generation sequencing
PB: peripheral blood
RT-PCR: reverse transcription-polymerase chain reaction
SMM: smoldering multiple myeloma
